# Southern Illinois Medical Association

**Published:** 1880-02

**Authors:** 


SOUTHERN ILLINOIS MEDICAL ASSOCIATION.—The recent annual meeting of this society appears to have been unusually well attended, and was an occasion of much interest and profit to its members. Drs. Hughes, Engleman and others were present from St. Louis, and addressed the meeting. The Southern Illinois Medical Association embraces a large territory in the Southwestern part of the State, and its members will doubtless give a cordial reception to the State Medical Society, which holds its next annual meeting at Belleville, on the third Tuesday in May next.

DR. WM. T. BELFIELD, of this city, is making preparations with a view to spending several months in Vienna, engaged in the special study of pathology. While there, he will be the special correspondent of the CHICAGO MEDICAL JOURNAL AND EXAMINER, and we shall take pleasure in laying before our readers the monthly labor of his ready pen.

